# Universal adaptive optics for microscopy through embedded neural network control

**DOI:** 10.1038/s41377-023-01297-x

**Published:** 2023-11-13

**Authors:** Qi Hu, Martin Hailstone, Jingyu Wang, Matthew Wincott, Danail Stoychev, Huriye Atilgan, Dalia Gala, Tai Chaiamarit, Richard M. Parton, Jacopo Antonello, Adam M. Packer, Ilan Davis, Martin J. Booth

**Affiliations:** 1 Department of Engineering Science, University of Oxford, Oxford, UK; 2 Department of Biochemistry, University of Oxford, Oxford, UK; 3 Department of Physiology, Anatomy, and Genetics, University of Oxford, Oxford, UK

**Keywords:** Adaptive optics, Multiphoton microscopy, Wide-field fluorescence microscopy

## Abstract

The resolution and contrast of microscope imaging is often affected by aberrations introduced by imperfect optical systems and inhomogeneous refractive structures in specimens. Adaptive optics (AO) compensates these aberrations and restores diffraction limited performance. A wide range of AO solutions have been introduced, often tailored to a specific microscope type or application. Until now, a universal AO solution – one that can be readily transferred between microscope modalities – has not been deployed. We propose versatile and fast aberration correction using a physics-based machine learning assisted wavefront-sensorless AO control (MLAO) method. Unlike previous ML methods, we used a specially constructed neural network (NN) architecture, designed using physical understanding of the general microscope image formation, that was embedded in the control loop of different microscope systems. The approach means that not only is the resulting NN orders of magnitude simpler than previous NN methods, but the concept is translatable across microscope modalities. We demonstrated the method on a two-photon, a three-photon and a widefield three-dimensional (3D) structured illumination microscope. Results showed that the method outperformed commonly-used modal-based sensorless AO methods. We also showed that our ML-based method was robust in a range of challenging imaging conditions, such as 3D sample structures, specimen motion, low signal to noise ratio and activity-induced fluorescence fluctuations. Moreover, as the bespoke architecture encapsulated physical understanding of the imaging process, the internal NN configuration was no-longer a “black box”, but provided physical insights on internal workings, which could influence future designs.

## Introduction

The imaging quality of high-resolution optical microscopes is often detrimentally affected by aberrations which result in compromised scientific information in the images. These aberrations can arise from imperfections in the optical design of the microscope, but are most commonly due to inhomogeneous refractive index structures within the specimen. Adaptive optics (AO) has been built into many microscopes, restoring image quality through aberration correction by reconfigurable elements, such as deformable mirrors (DMs) or liquid crystal spatial light modulators (LC-SLMs)^1–6^. Applications of AO-enabled microscopes have ranged from deep tissue imaging in multiphoton microscopy through to the ultra-high resolution required for optical nanoscopy. This range of applications has led to a wide variety of AO solutions that have invariably been tailored to a specific microscope modality or application.

There are two main classes AO operation: in one case, a wavefront sensor measures aberrations; in the other case, aberrations are inferred from images – so called “wavefront sensorless AO”, or “sensorless AO” for short. For operations with a wavefront sensor, phase aberrations are measured directly by wavefront sensors such as a Shack-Hartmann sensor^7,8^ or an interferometer^9,10^. Such operations are direct and fast but also have intrinsic disadvantages such as requiring a complex optical design and suffering from non-common path errors. Furthermore, such wavefront sensors often have limitations and are less versatile. For example, an interferometer requires a coherent source and all such methods suffer from problems due to out-of-focus light. On the other hand, sensorless AO methods normally function with a simpler optical design and thus are more easily adaptable for a wide range of imaging applications. However, sensorless AO methods are based on iterative deductions of phase aberrations and thus tend to be more time consuming; this is coupled with repeated and prolonged sample exposures, which inevitably lead to photo-damage or motion related errors.

There have been many developments in AO technology, and in particular sensorless AO methods. Conventionally, sensorless AO operates based on the principle that the optimal image quality corresponds to the best aberration correction^11,12^. A suitably defined metric, such as the total signal intensity^13–23^ or a spatial frequency based sharpness metric^24–28^, is used to quantify the image quality. Phase is modulated by the AO while this quality metric reading is measured and optimised. There have been discussions on how the phase should be modulated^11,29,30^ and how the optimisation algorithm should be designed^19,31–33^. However, as mentioned before, such “conventional” sensorless AO methods depend on iterative optimisation of a scalar metric, where all image information is condensed into a single value, and the optimisation process is usually through mode by mode adjustment. Such methods were thus not the most efficient approach to solving this multi-dimensional optimisation problem and the effective range of correction was limited. While a higher dimensional metric was considered to extract more information from images^34^, the optimisation of such a vector metric was not straightforward.

While the utility of each of these conventional sensorless AO methods has been demonstrated separately, each method had been defined for a particular microscope type and application. Until now, no such AO solution has been introduced that can be universally transferred between microscope modalities and applications.

We propose in this article a new approach to sensorless AO (named as MLAO) that addresses the limitations of previous methods and provides a route to a universal AO solution that is applicable to any form of microscopy. This solution is constructed around a physics-based machine learning (ML) framework that incorporates novel neural network (NN) architectures with carefully crafted training procedures, in addition to data pre-processing that is informed by knowledge of the image formation process of the microscope. The resulting NN is embedded into the control of the microscope, improving the efficiency and range of sensorless AO estimation beyond that possible with conventional methods. This approach delivers versatile aberration measurement and correction that can be adapted to the application, such as the correction of different types of aberration, over an increased range of aberration size, across different microscope modalities and specimens.

In recent years, machine learning (ML) has been trialed in AO for its great computational capability to extract and process information. However, many of these approaches required access to point spread functions (PSFs) or experimentally acquired bead images;^35–41^ these requirements limited the translatability of these methods to a wider range of applications. Reinforcement learning was applied to correct for phase aberrations when imaging non point-like objects;^42^ however, the method still involved iterative corrections and was not advantageous in terms of its correction efficiency, accuracy and correction working range compared to conventional sensorless AO algorithms. Untrained neural networks (NN) were used to determine wavefront phase and were demonstrated on non point-like objects;^43,44^ however, such methods were reported to normally require a few minutes of network convergence, which limits their potential in live imaging applications.

Our new approach differs considerably from previous ML assisted aberration estimation, as previous methods mostly employed standard deep NN architectures that used raw images as the input data. Our method builds upon physical knowledge of the imaging process and is designed around the abilities of the AO to introduce aberration biases, which improve the information content of the NN input data. This approach means that the resulting NN is orders of magnitude simpler, in terms of trainable parameters, than previous NN methods (See Table S1 in supplemental document). Furthermore, our method is readily translatable across microscope modalities. As NN training is carried out on a synthetic data set, adaptation for a different modality simply requires regeneration of the image data using a new imaging model. The NN architecture and training process are otherwise similar.

To illustrate the versatility of this concept, we have demonstrated the method on three different types of fluorescence microscopes with different forms of AO corrector: a two-photon (2-P) microscope using a SLM, a three-photon (3-P) intravital microscope using a DM, and a widefield three dimensional (3-D) structured illumination microscope (SIM) using a DM. In all cases, we showed that the new method outperformed commonly used conventional sensorless AO methods. The results further showed that the ML-based method was robust in a range of challenging imaging conditions, such as specimen motion, low signal to noise ratio (SNR), and fluorescence fluctuations. Moreover, as the bespoke architecture encapsulated into its design physical understanding of the imaging process, there was a link between the weights in the trained NN and physical properties of the imaging process. This means that the internal NN configuration needs no-longer to be considered as a “black box”, but can be used to provide physical insights on internal workings and how information about aberrations is encoded into images.

## Concept and implementation

**Fig. 1:**
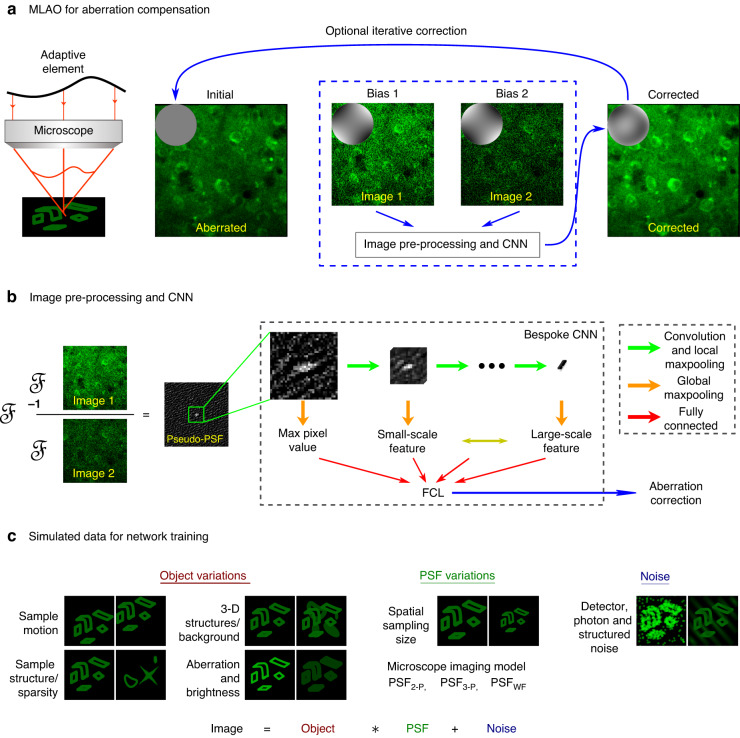
The MLAO concept. **a** Overview of the AO correction process. A minimum of two bias aberrations were introduced by the adaptive element; corresponding images of the same field were captured. The images were passed to the MLAO estimator, which determined the Zernike coefficients for correction. The correction speed was limited only by the speed of image acquisition, not by computation. Further correction could optionally be implemented through iteration. **b** Image pre-processing and NN architecture. Images were pre-processed to compute pseudo-PSFs, which were predominantly independent of specimen structure. ℱ and ℱ^-1^ represent the forward and inverse Fourier transform, respectively. A central cropped region of the pseudo-PSF images was used as the input to a convolutional neural network (CNN). The CNN was designed and trained specifically for aberration determination. The output from the overall network was the correction coefficients for the Zernike modes. The NN architecture was such that the convolutional layer outputs could be correlated with spatial scales of the aberration effects on the pseudo-PSFs and hence the imaging process. Hence, the distribution of weights in the fully connected layer (FCL) of the network had physical relevance. **c** Training data generation. A range of image variations were included in the synthetic data set for NN training to cope with variations in real experimental scenarios. The data were a combination of artificial and real microscope images, chosen to model a wide range of realistic specimen structures. Images were created through convolution of specimen structures with an appropriate PSF, generated for the specific microscope modality, incorporating aberrations. Details of the training data synthesis and data augmentation can be found in section 2 of the supplemental document

The overall MLAO concept is illustrated in Fig. 1. The experimental application follows closely the concept of modal sensorless AO, whereby a sequence of images are taken, each with a different bias aberration applied using the adaptive element. The bias aberrations are a set of pre-defined phase modulation intentionally introduced into the system for phase diversity^11^. The set of images are then used as the input to the ML-enabled estimator, which replaces the previous conventional method of optimisation of an image quality metric. The estimated correction aberration is then applied to the adaptive element. If necessary, the process can be iterated for refined correction. The significant advantage of the new method is the way in which the estimator can more efficiently use image information to determine the aberration correction.

The concept has been designed in order to achieve particular capabilities that extends beyond those of conventional sensorless AO. The new method should ideally achieve more efficient aberration estimation from fewer images, to reduce time and exposure of measurement. It should operate over a larger range of aberration amplitudes, compared to previous methods. A particular estimator should be robust to variations between similar microscopes and the concept should be translatable across different microscope types and applications. From a practical perspective, it is also important that training can be performed on synthetic data, as it would be impractical to obtain the vast data set necessary for training from experimentally obtained images.

An essential step towards efficient use of image data is the image pre-processing before they are presented to the NN. Rather than taking raw image data as the inputs, the NN receives pre-processed data calculated from pairs of biased images, which we term a “pseudo-PSF”, as shown in Fig. 1 and explained in the methods section. This pseudo-PSF contains information about the input aberration and is mostly independent of the unknown specimen structure. By removing the specimen information at this stage, we can reduce the demands on the subsequent NN, hence vastly simplifying the architecture required to retrieve the aberration information. In all cases used in this paper, each pseudo-PSF was calculated from a pair of images obtained with equal magnitude, but opposite sign, bias aberration.

As most of the useful information related to aberrations was contained within the central pixels of the pseudo-PSF, a region of 32 × 32 pixels was extracted as the input to the NN. The first section of the NN was a bespoke convolutional layer that was designed to extract information from the inputs at different spatial scales. The outputs from the convolutional layer were then provided to a fully connected layer, which was connected to the output layer. Full details of the NN design are provided in the methods and the supplementary document. This architecture—rather unusually—provided a link between the physical effects of aberrations on the imaging process and the mechanisms within the NN, specifically through the weights at the output of the first fully connected layer.

NN training was performed using a diverse set of synthesised training data. These images were calculated using an appropriate model of the microscope imaging process in the presence of aberrations. Images were synthesised by convolutions of specimen structures with a PSF, incorporating various likely experimental uncertainties and noise sources. The specimens consisted of a range of artificial and realistic objects. Full details of training data synthesis and data augmentation are provided in the methods and section 2 of supplemental information.

This versatile concept could accommodate different aberration biasing strategies. Conventional modal sensorless AO methods typically required a minimum of 2 *N* + 1 biased images to estimate *N* aberration modes^19^. However, the MLAO method has the ability to extract more information out of the images, such that aberrations could be estimated with as few as two images, although more biased images could provide better-conditioned information. In general, we defined methods that used *M* differently biased images to estimate *N* Zernike modes for aberration correction. The input layer of the NN was adjusted to accommodate the *M* image inputs for each method. Out of the many possibilities, we chose to illustrate the performance using two biasing schemes: one using a single bias mode (astigmatism, Noll index^45^ i = 5) and one using all *N* modes that were being corrected. In the first case, we used either two or four images (*M* = 2 or 4) each with different astigmatism bias amplitude. We refer to these methods as *ast2 MLAO* or *ast4 MLAO*. Astigmatism was chosen as the most effective bias mode (see supplementary document, section 7). In the second case, biased images were obtained for all modes being estimated (*M* = 2 *N* or 4 *N*); this type is referred to in this paper as *2* *N MLAO* or *4* *N MLAO*. For a complete list of the settings for each demonstration, please refer to Table S2 in the supplemental document.

## Results

In order to show its broad application, the MLAO method was demonstrated in three different forms of microscopy: 2-P and 3-P scanning microscopy and widefield 3-D SIM. This enabled testing in different applications to examine its performance coping with different realistic imaging scenarios.

The MLAO methods were compared to two widely used conventional modal based sensorless AO methods (labelled as *2* *N* + *1 conv* and *3* *N conv*). The *2* *N* + *1 conv* method used two biased images per modulation mode and an additional zero biased image to determine phase correction consisting *N* modes simultaneously. The *3* *N conv* method used three images per modulation mode (two biased and one unbiased images) and determined the coefficients of the modes sequentially. For both methods, the bias size was chosen to be *±*1 rad for each mode. A suitable metric was selected to quantify the image quality.

For each mode, the coefficients were optimised by maximising the quality metric of the corresponding images using a parabolic fitting algorithm. When used in 2-P and 3-P demonstrations, the total fluorescence intensity metric was optimised. For the widefield 3-D SIM microscope, a Fourier based metric was optimised^46^. For the details of the two conventional methods, please refer to^19,31^.

Different functions were defined as optimisation metrics for the conventional AO methods, and also to assist quantifiable comparisons of image quality improvement for the MLAO methods. These were defined as an intensity based metric y_I_, a Fourier based metric y_F_, a sharpness metric y_S_ and a Fourier threshold based metric y_T_. Details are provided in the methods section.

### Two-photon microscopy

A range of method validations were performed on a 2-P microscope that incorporated a SLM as the adaptive correction element, including imaging bead ensembles and extended specimen structures. The experimental set-up of the 2-P system was included in Figure S9a in the supplemental document. In order to obtain controlled and quantitative comparisons between different AO methods, the SLM was used to both introduce and correct aberrations. This enabled statistical analysis of MLAO performance with known input aberrations. System aberrations were first corrected using a beads sample before carrying out further experiments.

**Fig. 2:**
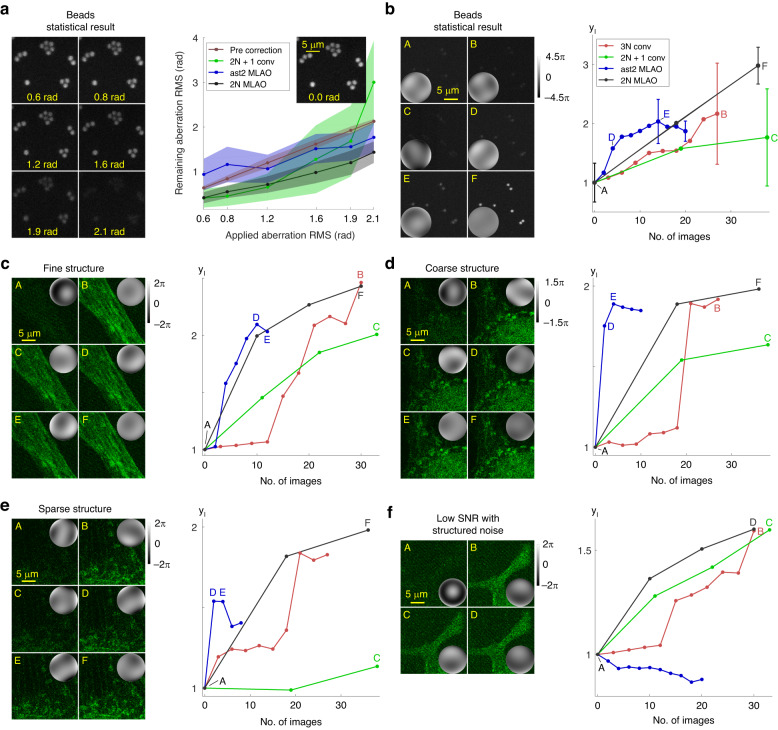
Comparative performance of MLAO methods in a 2-P microscope. **a** Residual aberration after one correction cycle for three methods. Points show the mean and the shaded area indicates the standard deviations (SDs) of aberration distributions. The images show an example field of view (FOV) when different amounts of a random aberration were introduced. **b**–**f** show the normalised intensity metric (y_I_) as a proxy for correction quality, against the number of images used for multiple iterations of correction when random aberrations were introduced. In (**b**), an ensemble of ten random aberrations were corrected, imaging over the same FOV. Error bars on the plot showed the SDs of the fluorescence intensity before and after correction. **c**–**f** show specific corrections imaging microtubules of BPAE cells, illustrating performance for different specimen structures and imaging conditions. The images were acquired before and after correction through the different methods (as marked on the metric plots). Insets on the images show residual wavefronts after correction for each image. The grayscale colorbars show phase in radians

We performed a statistical analysis to assess how MLAO algorithms (*ast2 MLAO* and *2* *N MLAO*) performed in various experimental conditions compared to conventional algorithms (*2* *N* + *1 conv* and *3* *N conv*). Experiments were conducted on fixed beads samples (Fig. 2a, b), and Bovine Pulmonary Artery Endothelial (BPAE) cells (FluoCells^TM^ Prepared Slide #1) (Fig. 2c–f). Dependent on the experiment, either *N* = 5 or *N* = 9 Zernike modes were estimated (see Table S2 in Supplemental document for details).

#### Statistical performance analysis

Figure 2a, b showed statistical comparisons of the different correction methods. Figure 2a displayed the residual aberrations gathered from twenty experiments, each consisting of one correction cycle from random initial aberrations including five Zernike modes. If the remaining aberration is below the pre-correction value, then the method provides effective aberration correction. A wide shaded area indicated inconsistent and less reliable correction. The results show that when correcting small aberrations with root mean square (RMS) = 0.63 to 1.19 rad, *2* *N MLAO* performed similarly to *2* *N* + *1 conv*. Between RMS = 1.19 to 1.92 rad, *2* *N MLAO* corrected more accurately (lower mean aberration) and also more reliably (smaller error range). For large aberrations above RMS = 2.12 rad, *2* *N* + *1 conv* completely failed, whereas the MLAO methods still improved aberration correction. *ast2 MLAO* had poor performance at small aberrations (RMS = 0.63 to 0.84 rad) but provided reasonable correction for large aberrations (RMS = 1.92 to 2.12 rad). However, it is important to note that *ast2 MLAO* required only two images for each correction cycle, far fewer that the ten and eleven images required respectively for *2* *N MLAO* and *2* *N* + *1 conv*.

Figure 2b displayed the mean value of metric y_I_ from ten experiments against the number of images acquired during multiple iterations of the different correction methods. The corrected aberrations consisted of nine Zernike modes. It was shown that *ast2 MLAO* corrects the fastest initially when the input aberration is large but converges to a moderate signal level, which indicates only partial correction of the aberration. *2* *N MLAO* corrects more quickly and to a higher level than the conventional algorithms. The narrower error bars for both MLAO algorithms at the end of the correction process indicate that they are more reliable than the two conventional methods.

#### Correction on extended specimen structures

Figure 2c–f showed experimental results when imaging microtubules of BPAE cells. Specimen regions were chosen to illustrate performance on different structures: (c) contained mainly aligned fine fibrous structures; (d) contained some large scale structures (bottom right); (e) contained fine and sparse features. For (f) we intentionally reduced illumination laser power and increased detector gain to simulate an imaging scenario with very low SNR. The images showed structured noise at the background, which could pose a challenge to estimation performance. A large randomly generated aberration (RMS = 2.12 to 2.23 rad) consisting of five (c and f) or nine (d and e) Zernike modes was used as the input aberration.

In (c), (d) and (e), *ast2 MLAO* corrected the fastest initially when the aberration was large but converged to a moderate level of correction. *2* *N MLAO* corrected faster in general than the conventional methods and converged to a higher level of correction. In (f) when SNR was poor and structured noise was present, *ast2 MLAO* failed to correct while *2* *N MLAO* continued to perform consistently.

### Three-photon intravital microscopy

Three-photon microscopy of neural tissue imaging is a particular challenge for sensorless AO, due to the inherently low fluorescence signal levels. While this could be alleviated by averaging over time, problems are created due to specimen motion. Further challenges are posed for functional imaging, due to the time dependence of emission from ion or voltage sensitive dyes. The demonstrations here show the robustness of the new MLAO methods in experimental scenarios where the conventional methods were not effective. Importantly, the MLAO methods were able to perform effective correction based on a small number of low SNR image frames without averaging.

**Fig. 3:**
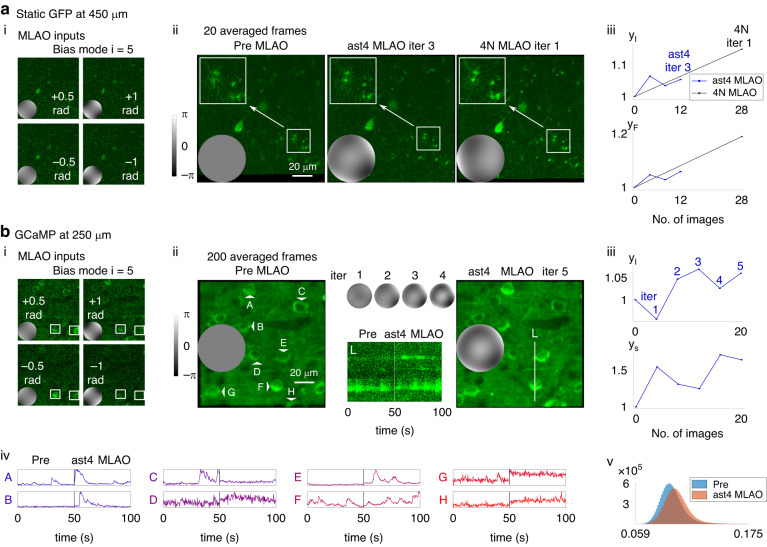
Aberration correction in three-photon microscopy of live mouse brains: (**a**) GFP-labelled cells at depth 450 µm and (**b**) functional activity of GCaMP-labelled cells at 250 µm. Wavefronts inserted into the figures showed the phase modulations applied by the DM at the relevant steps; the common scale for each set of results is indicated by the grayscale bars in (**a**) and (**b**). **a**–i Shows on the left example single-frame images used in correction with the corresponding bias modes as insets; these were the image inputs to *ast4 MLAO*. For *4* *N MLAO*, six more bias modes and thus 24 more images were also used in each iteration. Three images at the central (**a**-ii) are shown averaged from 20 frames after motion correction. The rectangular boxes highlight regions of interest for comparison. The plots on the right (**a**-iii) show the intensity metric (y_I_) and the Fourier metric (y_F_), respectively, calculated from single image frames, against the number of images acquired for three correction iterations of *ast4 MLAO* and one correction iteration of *4* *N MLAO*. **b**–i Shows on the left example single-frame images used as inputs to the *ast4 MLAO* correction with the corresponding bias modes as insets. White squares highlight two cells for comparison to show the fluorescence fluctuations over time due to neural activity. The central (**b**-ii) shows respectively before and after *ast4 MLAO* correction through five iterations (iter 1–5), 200 frame averages after motion correction. The time traces were taken from the marked line L. The plots on the right (**b**-iii) show the intensity metric (y_I_) and the sharpness metric (y_S_), respectively, calculated from single image frames, against the number of images acquired for five iterations *ast4 MLAO*. The lower panel (**b**-iv) shows the calcium activity of 8 cells (A-H marked on the averaged image). The lower right plot (**b**-v) shows the histograms of the 200 frames collected before and after *ast4 MLAO* corrections. The pixel values were normalised between 0 and 1

The experimental set-up of the 3-P system is shown in Figure S9b in the supplemental document. The microscope used an electromagnetic DM for aberration biasing and correction. Two MLAO methods, *ast4 MLAO* and *4* *N MLAO*, were used to correct aberrations by using single frame images as inputs. In each case, more input frames were chosen than in the 2-P demonstrations, in order to cope with the lower SNR. The NNs were trained to estimate *N* = 7 Zernike modes. Two types of mice were used to perform live brain imaging of green fluorescent protein (GFP) labelled cells (Fig. 3a) and functional imaging in GCaMP-expressing neurons (Fig. 3b). In Fig. 3a, results were collected at 450 µm depth and power at sample was 32 mW. In Fig. 3b, imaging was at 250 µm depth and power at sample was 19 mW. Further 3-P results were included in the section 9 of supplemental document. For the details of the sample preparation, please refer to section 10B in supplemental document.

Figure 3a-iii shows plots of the metrics y_I_ and y_F_ as proxies for correction quality when imaging GFP labelled cells. Both *ast4 MLAO* and *4* *N MLAO* networks successfully improved the imaging quality. Similar to the *ast2 MLAO* results in the 2-P demonstrations, *ast4 MLAO* corrected more quickly at first, but converged to a lower correction level. In contrast, *4* *N MLAO* performed better overall correction, but required more images. Panel (a-ii) show averaged images in which blurry processes previously hidden below the noise level are revealed and get clearer through MLAO correction (as highlighted in the white rectangles). The example biased images shown in the left panel (a-i) provide an indication of the low raw-data SNR that the MLAO method can successfully use.

Figure 3b shows results from imaging calcium activity in a live mouse. Panel (b-iii)showed that the *ast4 MLAO* method successfully improved image quality despite the low SNR and fluorescence fluctuations of the sample. From both (b-ii) time traces of line 1 and (b-iv) cells A-H, it could be clearly seen that after corrections, signals were increased. The *4* *N MLAO* method failed to correct in this experimental scenario (results not shown). We will discuss the likely hypotheses for this in the discussion section.

The fluctuating fluorescence levels due to neural activity mean that conventional metrics would not be effective in sensorless AO optimisation processes. This is illustrated in Fig. 3b-iii, where it can be seen that no single metric can accurately reflect the image quality during the process of *ast4 MLAO* correction. These observations illustrate the advantages of MLAO methods, as their optimisation process did not rely on any single scalar metric.

### Widefield 3-D structured illumination microscopy

The architecture of the NN was conceived so that it would be translatable to different forms of microscopy. In order to illustrate this versatility, and to complement to the previously shown 2-P and 3-P laser scanning systems, we applied MLAO to a widefield method. The 3D SIM microscope included multiple lasers and fluorescence detection channels and an electromagnetic DM as the correction element. Structured illumination patterns were introduced using a focal plane SLM. The detailed experimental set-up was included in Fig. S9c in the supplemental document. Further widefield results were included in the section 9 of supplemental document.

Without AO, 3D SIM reconstruction suffers artefacts caused by aberrations. Since typical specimens contain 3D structures, the lack of optical sectioning in widefield imaging means that the aberration correction process can be affected by out of focus light. As total intensity metrics are not suitable for conventional AO algorithms in widefield imaging, Fourier based sharpness metrics have often been used. However, such metrics depend on the frequency components of the specimen structure^34^. In particular, emission from out of focus planes can also affect the sensitivity and accuracy of correction. However, the NN based MLAO methods were designed and trained to mitigate against the effects of the sample structures and out of focus light.

**Fig. 4:**
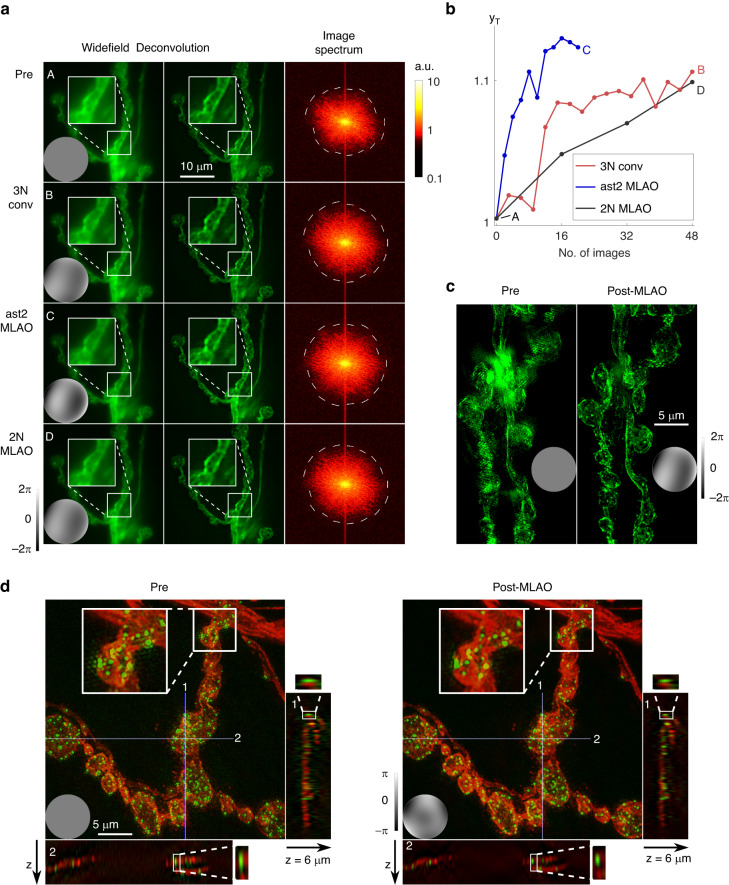
Aberration correction in a widefield 3-D structured illumination microscope (SIM). **a** Widefield images acquired A before and B-D after correction through different methods (as marked on the metric plot (**b**)). The second column shows corresponding deconvolved widefield images. The third column shows corresponding image spectra of the first column widefield images displayed in a logarithmic scale (as shown in the colorbar); dashed lines show the threshold where signal falls below the noise level. **b** The frequency threshold metric y_T_ against the number of images, for two iterations of *3* *N conv*, ten iterations of *ast2 MLAO* and three iterations of *2* *N MLAO*. 3-D projections of 3-D reconstructed SIM image stack of (**c**) 10 µm and (**d**) 6 µm when by-passing AO and after five iterations of *2* *N MLAO* correction. **d** Square inserts show zoomed-in region for comparison. x-z and y-z sections are shown through lines 1 and 2. Insets to (**a**, **c**, **d**) show wavefronts corrected by the DM for each image acquisition; phase is shown on the adjacent scale bar

Figure 4 shows results from two NN-based methods *ast2 MLAO* and *2* *N MLAO* compared to the conventional algorithm *3* *N conv*, which used the y_S_ metric. Sensorless AO was implemented using widefield images as the input (Fig. 4a, b). Previous work showed that aberration correction within the passband of conventional widefield images is sufficient for correcting high frequency components of SIM reconstruction^47^. The correction settings thus obtained by the *2* *N MLAO* method were then applied to super-resolution 3D SIM operation (Fig. 4c, d). *N* = 8 Zernike modes were involved in the aberration determination. The specimen was a multiple labelled *Drosophila* larval neuromuscular junction (NMJ). For the details of the sample preparation, please refer to section 10B in supplemental document.

Figure 4b showed that *ast2 MLAO* corrected most quickly; *2* *N MLAO* corrected similarly as *3* *N MLAO* and were less effective. Figure 4a showed the effectiveness of correction on raw and deconvolved widefield images. The third column showed the changes in image spectrum of the widefield images after correction. The dashed line shows a threshold where signal falls below the noise level. It can be seen that all three methods increased high frequency content compared to (A) before AO correction. Figure 4c, d showed the images after 3D SIM reconstruction. It can be clearly seen that when by-passing AO (left hand side), there were strong artefacts due to aberrations. After correcting using five iterations of *2* *N MLAO*, artefacts were suppressed and z-resolution was improved (see sections through line 1 and 2 in Fig. 4d) Comparing the widefield images in (a) and SIM reconstructed images in (c), it is notable that the difference before and after correction of the widefield images is not large. The reason for this is that small amounts of aberration that have little effect on widefield images can have significant effects on super-resolution images.

## Discussion

The power and simplicity of the MLAO method arise mainly from a combination of three aspects: the pre-processing of image data, the bespoke NN architecture, and the definition of the training data set. All of these aspects are informed by physical and mathematical principles of image formation. This forms a contrast with many other data-driven deep learning approaches, where complex NNs are trained using vast amount of acquired data.

The calculation of the pseudo-PSF from pair of biased images (as shown in Fig. 1c and elaborated in the “Methods”) acts to remove most of the effects of unknown specimen structure from the input data. The information contained within the pseudo-PSF encodes indirectly how aberrations affect the imaging PSF (see Fig. S2 in the supplemental document for more details). There is a spatial correspondence between a pixel in the pseudo-PSF and the PSF itself. Hence, spatial correlations across the pseudo-PSF relate to spatial effects of aberrations on the images.

The set of pseudo-PSFs forms the input to the convolutional layers of the NN. The masks in each convolutional layer probe, in effect, different scales across the pseudo-PSF. Hence, one can attribute a correspondence between the output of these layers and the effects aberrations have over different physical scales in the image. Such phenomena are heuristically demonstrated in section 3 of the supplementary information. By extracting relevant weight connections from inside the NN, we can observe embedded physical interpretations of how the machine learned to process aberration information contained in images.

To illustrate this, we extracted from the trained NN the weights between the layer embedding physical interpretations and the next fully connected layer (marked by the red arrows in Fig. 1a and the red arrow enclosed by the dashed square in Fig. S1 in the supplemental document). Going down the convolutional layers, the scale of probed features increases from a single pixel, through small scale features, up to large scale features (as explained in section 4 of the supplemental document). The RMS values of the weights from each convolutional layer are shown in Table 1, where the data are shown for the ensembles of the two classes of MLAO networks used in this paper, *astX MLAO* and *XN MLAO* (where *X* = 2 or 4). A full breakdown is provided in the Figure S4 of the supplementary document.

**Table 1:** The RMS of the weight distributions extracted from different convolutional layers of the two classes of trained CNNs, astX MLAO and XN MLAO

Layer	1	2	3	4	5
*astX MLAO*	0.23	0.19	0.17	0.18	0.23
*XN MLAO*	0.39	0.14	0.15	0.13	0.20

The values shown are calculated from the ensemble of corresponding layers from all CNNs of the given class

The largest weight variation was in the first layer in the *XN MLAO* NN, which indicates that this algorithm extracts more information from the single pixel detail than from larger scale correlations. In contrast, *astX MLAO* assigns weights more evenly across all layers. As explained in the supplementary document, the single pixel extraction from the pseudo-PSF is related to the Strehl ratio of the PSF and the intensity information of the images in non-linear systems. Hence, it is expected that the *XN MLAO* NN, which uses as similar set of bias aberrations to the conventional method, would learn as part of its operation similar behaviour to the conventional algorithm. The same phenomena can also explain why in 3-P GCaMP imaging of neural activity *astX MLAO* was less affected by the fluorescence fluctuations than *XN MLAO*, as *astX MLAO* relies less on overall fluorescence intensity changes. Similarly, in widefield imaging *astX MLAO* was more effective at extracting PSF variations than *XN MLAO* as the overall fluorescence intensity did not change with aberrations in single-photon imaging. Conversely, *astX MLAO* generally performed worse than *XN MLAO* in 2-P imaging when structured noise present, as *astX MLAO* used fewer images and hence had access to less detectable intensity variations than *XN MLAO*. The fact that *astX MLAO* had access to less well-conditioned image information may also explain why in general it was able to correct aberrations to a lower final level than *XN MLAO*.

## Conclusion

The MLAO methods achieved the aims explained at the outset. They provided more efficient aberration correction with fewer images over a larger range, reducing time required and specimen exposure. The training procedure, which was based on synthesised data, ensured that the AO correction was robust to uncertainty in microscope properties, the presence of noise, and variations in specimen structure. The concept was translatable across different microscope modalities, simply requiring training using a revised imaging model.

The new methods used NN architectures that are orders of magnitude simpler, in terms of trainable parameters, than in previous similar work (see supplementary information, section 6). This vast simplification was achieved through pre-processing of data to remove most of the effects of unknown specimen structure. The physics-informed design of the NN also meant that – unusually for most NN applications – the learned weights inside the network provided indications of the physical information used by the network. This provides constructive feedback that can inform future AO system designs and the basis for extension of the MLAO concept to more demanding tasks in microscopy and other imaging applications.

## Methods

### Image pre-processing

Image data were pre-processed before being used by the NN, in order to remove effects of the unknown specimen structure. The resulting “pseudo-PSFs” were better conditioned for the extraction of aberration information, independently of the specimen. The image formation can be modelled as a convolution between specimen fluorescence distribution and an intensity PSF. The AO introduced pre-chosen bias aberrations, so that multiple images with different PSFs could be acquired over the same FOV. Mathematically, this process can be expressed as

1 $$\begin{array}{c}{I}_{1}=O* {f}_{1}+{\delta }_{1}\\ {I}_{2}=O* {f}_{2}+{\delta }_{2}\end{array}$$

where *I*_1_ and *I*_2_ were the images acquired with two different PSFs *f*_1_ and *f*_2_ for the same unknown specimen structure *O*. ${\delta }_{1}$ and ${\delta }_{2}$ represent combined background and noise in each image. In order to remove (or at least reduce) the effects of specimen structures, we defined the pseudo-PSF as

$${\rm{pseudo}}-{\rm{PSF}}={{\mathscr{F}}}^{-1}\left[\frac{{\mathscr{F}}\left({I}_{1}\right)}{{\mathscr{F}}\left({I}_{2}\right)}\right]={{\mathscr{F}}}^{-1}\left[\frac{{\mathscr{F}}\left(O* {f}_{1}+{\delta }_{1}\right)}{{\mathscr{F}}\left(O* {f}_{2}+{\delta }_{2}\right)}\right]={{\mathscr{F}}}^{-1}\left[\frac{{\mathscr{F}}\left(O\right)\times{\mathscr{F}}\left({f}_{1}\right)+{\mathscr{F}}\left({\delta }_{1}\right)}{{\mathscr{F}}\left(O\right)\times {\mathscr{F}}\left({f}_{2}\right)+{\mathscr{F}}\left({\delta }_{2}\right)}\right]$$

where ℱ was the 2D Fourier transform and ℱ^-1^ was its inverse (see Fig. 1c). The term “pseudo-PSF” was chosen as the function was defined in the same variable space as a PSF, although it is not used directly in any imaging process. A similar computational process was shown elsewhere for different applications using defocussed images^48^. Assuming the noise is small enough to be neglected

2 $${\rm{pseudo}}-{\rm{PSF}}={{\mathscr{F}}}^{-1}\left[\frac{{\mathscr{F}}\left({I}_{1}\right)}{{\mathscr{F}}\left({I}_{2}\right)}\right]\approx {{\mathscr{F}}}^{-1}\left[\frac{{\mathscr{F}}\left({f}_{1}\right)}{{\mathscr{F}}\left({f}_{2}\right)}\right]$$

There is an implicit assumption here that there are no zeroes in the object spectrum ℱ (*O*) or the optical transfer function ℱ (*f*_2_). In practice, it was found that a small non-zero value of ℱ (${\delta }_{2}$ mitigated against any problems caused by this. Furthermore, although structured noise was present in the pseudo-PSFs (see e.g. Fig. S1 in the supplemental document), it was found that this did not detrimentally affect data extraction through the subsequent NN. As a further mitigation, we calculated pairs of pseudo-PSFs from pairs of biased input images by swapping the order from (*f*_1_*, f*_2_) for the first pseudo-PSF to (*f*_2_*, f*_1_) for the second.

Example pseudo-PSFs are shown in Fig. S1, S2 in the Supplemental document. As most information was contained within the central region, to ensure more efficient computation, we cropped the central region (32 × 32 pixels) of the pseudo- PSFs to be used as the input to the NN. Dependent upon the MLAO algorithm, the input to the NN would consist of a single pair of cropped pseudo-PSFs, or multiple pairs corresponding to the multiple pairs of bias aberrations applied in different modes.

### Neural network training

To estimate phase aberrations from pseudo-PSFs, a convolutional based neural network was designed incorporating physical understanding of the imaging process and was trained through supervised learning. Synthetic data were used for training and the trained networks were then tested on real AO microscopes. For each imaging modality (i.e. 2-P, 3-P and widefield), a separate training dataset was generated, with the imaging model and parameters adjusted for different applications. For the details of neural network architecture and synthetic training data generation, please see section 1 and 2 of the supplementary information.

### Image quality metrics

Different image quality metrics were defined for use as the basis for optimisation in conventional sensorless AO methods and as proxies to quantify the level of aberration correction. y_I_ is an intensity based metric and can be used in non-linear imaging systems. It is defined as

$${y}_{I}=\mathop{\sum }\limits_{i}^{l}T\left(i\right)$$

where *T* (*i*) is a flattened array of image *I*(*x*) after sorting pixel values in descending order (indexed *i*). y_I_ is computed to sum only the first *l* pixel values to provide a fair quantitative intensity variation analysis when imaging sparse samples. *l* was adjusted for different experiments depending on the density of the sample structures and was chosen to be always larger than 200.

y_F_ is a Fourier based metric and provides an alternative aspect to the intensity metric. It is defined as

$${\rm{y}}_{\rm{F}}={\iint }_{{\rm{|}}f{\rm{|}} > 0.1{f}_{\min }}^{{\rm{|}}f{\rm{|}} < 0.6{f}_{\max }}\left|{\mathscr{F}}\left[I\left(x\right)\right]\right|d^2f$$

where ℱ[*I*(*x*)] is the 2D Fourier transform of image *I*(*x*) from *x* domain to *f* domain; *f*_*max*_ is the maximum frequency limit of the imaging system. The range 0.1 *f*_*max*_ < *| f |* < 0.6 *f*_*max*_ was selected such that most PSF related frequency information was included in the range.

y_S_ is a sharpness metric that can be used for optimisation in widefield systems, where the other metrics are not practical, or applications with fluorescence fluctuations. It is defined as

$${{\rm{y}}}_{{\rm{S}}}=\frac{{\iint }_{\left|f\right| > n{f}_{\max }}^{\left|f\right| < m{f}_{\max }}\left|{\mathscr{F}}\left[I\left(x\right)\right]\right|{d}^{2}f}{{\iint }_{\left|f\right| > 0}^{\left|f\right|\le n{f}_{\max }}\left|{\mathscr{F}}\left[I\left(x\right)\right]\right|{d}^{2}f}$$

where 1 *> m* > *n* > 0. This metric is defined as the ratio of higher to lower spatial frequency content, which is dependent upon aberration content, but independent of changes in overall brightness. *m* and *n* can be adjusted for different imaging sample structures such that the frequency components 0 *< | f* | ≤ *n f*_max_ contain mainly sample features and *n f*_max_ < *| f |* < *m f*_max_ captures mainly PSF sharpness. In Fig. 3b, *n* was chosen to be 0.05 and *m* was chosen to be 0.6.

y_T_ is a frequency threshold metric that can be used to analyse the image quality in widefield systems. It is defined as

$${{\rm{y}}}_{{\rm{T}}}=\frac{{\iint }_{0}^{2\pi }{f}_{T}(\theta ){\rm{d}}\theta }{{\iint }_{0}^{2\pi }{\rm{d}}\theta }$$

where *f*_*T*_ (*θ*) is the maximum frequency component for each angular segment *θ* such that *|FI* (*∀ f* < *f*_*T*_ (*θ*)*, θ*)*|* ⩾ *T*. *FI* (*f, θ*) = ℱ[*I*(*x*)] is the Fourier transform of image *I*(*x*) expressed in polar coordinates (*f, θ*). *T* is a threshold value such that *|FI* (*f, θ*)*|* < *T* can be considered as noise.

### Microscope implementations

Three microscopes were used to demonstrate and examine the MLAO method. The microscope implementations are briefly described here and fully elaborated in the supplementary document section 10 A.

In the home built 2-P system, a Newport-Spectra-Physics DeepSee femtosecond laser was used as the illumination with wavelength set at 850 nm. Light was modulated by a Hamamatsu spatial light modulator before passing through a water immersion objective lens with NA equals to 1.15 and reaching the sample plane.

A commercial Scientifica microscope system was used as the basis for our 3-P demonstration. In the 3-P system, a femtosecond laser passed through a pair of compressors and operated at 1300 nm. Light was modulated by a Mirao 52E deformable mirror before reaching a water dipping objective lens with NA equals to 0.8.

In the home built widefield 3D SIM system, two continuous wave lasers with wavelengths equal to 488 and 561 nm were used as the illumination. Light was modulated by a ALPAO 69 deformable mirror before reaching a water dipping objective lens with NA of 1.1.

### Image acquisition and processing

For 3-P imaging of live specimens, where motion was present, averaging was performed after inter-frame motion correction using TurboReg^49^. Time traces were taken from 200 raw frames captured at 4 Hz consecutively for each of the pre- and post-MLAO corrections.

For the widefield/SIM results, widefield images were processed where indicated using the Fiji iterative deconvolution 3-D plugin^50^. A PSF for deconvolution was first generated using the Fiji plugin Diffraction PSF 3-D with settings the same as the widefield microscope. For the deconvolution, the following settings were applied: Wiener filter gamma equals to 0; both x-y and z direction low pass filter pixels equal to 1; maximum number of iterations equals to 100; and the iteration terminates when mean delta is smaller than 0.01%.

The thresholds shown on the widefield image spectra were calculated by identifying the largest frequency in all x, y directions with image spectrum components higher than noise level. The noise level was identified by averaging the components of the high spectral frequency, i.e. at the four corners of the image spectrum. Starting from the lowest frequency, each angular and radial fragment was averaged and compared to the noise level. The largest component which was still above the noise level was traced on the image spectra by the dashed line and identified as the threshold.

The 3D-SIM reconstructions were extracted from image stacks where 15 image frames were collected per focal position using the SoftWorx package (Applied Precision)^51^. The projected images were obtained by summing frames at different z depths into an extended focus xy image.

### Supplementary information

Supplemental document

## Data Availability

The datasets generated during and/or analysed during the current study are available from the corresponding author on reasonable request.
