# Supplementary material for: Universal adaptive optics for microscopy through embedded neural network control
Source: Light Sci Appl. 2023 Nov 13;12:270. doi: 10.1038/s41377-023-01297-x (PMC10641083; doi:10.1038/s41377-023-01297-x)
Supplement: Supplementary file 1 — Supplemental document [file 41377_2023_1297_MOESM1_ESM.pdf]

Universal adaptive optics for microscopy through embedded neural network control: supplemental document

QI HU, MARTIN HAILSTONE, JINGYU WANG, MATTHEW WINCOTT, DANAIL STOYCHEV, HURIYE ATILGAN, DALIA GALA, TAI CHAIAMARIT, RICHARD M. PARTON, JACOPO ANTONELLO, ADAM M. PACKER, ILAN DAVIS, MARTIN J. BOOTH

1. MLAO PROCESS IN MICROSCOPE CONTROL AND CNN ARCHITECTURE

The machine learning adaptive optics (MLAO) neural network (NN) was implemented in a sensorless adaptive optics (AO) software package which was integrated into the microscope control system. One could choose to use either a MLAO or a conventional sensorless AO algorithm to conduct aberration correction. While training a NN for MLAO, we used a Python package - tensorflow-gpu which has a GPU requirements.[1]The implementation of the trained NN did not require a GPU. This sensorless AO software interacted with the AO control software and the image acquisition software. The sensorless AO software sent a signal to the AO control software to display the desired phase on the AO. It then controlled the image acquisition software and received an image from the software. The sensorless AO software then processed the images collected using the desired algorithm (MLAO or conventional algorithm) and the correction was then sent to the AO control software for aberration compensation. A final image was captured when the aberration correction process finished. The requirements for the image acquisition software and the AO control software could be flexible. Being constructed in this way, the MLAO package was mostly independent of the nature of the acquisition software used in each microscope.

The MLAO aberration estimation process consisted of two parts: image pre-processing to compute pseudo-PSFs from images and a CNN-based machine learning process for mode coefficient determination.

A stack of M images over the same field of view, each with a different pre-determined bias phase modulation, was used to calculate pseudo-PSFs according to the procedure in the methods section. The sequence of image pairs for pseudo-PSF generations was flexible as long as it was pre-defined and kept consistent during the network training. For all the demonstrations in this paper, M pseudo-PSFs were generated from M images where images with the same biases of the same magnitude and opposite signs were paired to generate a pair of pseudo-PSFs. It was observed and understood that most of the information was contained within the central region of the calculated pseudo-PSFs. [1] A central patch of 32×32 pixels was then cropped and used as the inputs to the CNN. Cropped pseudo-PSFs were processed by a sequence of convolutional layers (CL) with trainable 3×3 kernels, each followed by a local 2×2 max-pooling and thus the x and y sizes were reduced by half but the stack size was increased twice going down each CL. For the input pseudo-PSFs and each of the CL outputs, a global max-pooling was applied and concatenated into a fully connected layer (FCL). This concatenated FCL was connected to the next FCL containing 32 neurons, which in turn was connected to the output layer, which produced the coefficients of the N chosen Zernike modes. The activation functions were chosen to be tanh and linear (only for the last layer connection FCL 32 and the output). The regularizer used was L1L2,

[1]The process of calculating pseudo-PSFs can be interpreted as a deconvolution between two PSFs. Depending on the sampling size of the imaging system, most details of a deformed PSF typically occupy a central region of a few pixels. Most features of the pseudo-PSFs were thus captured within the central region.

the initializer was glorot-uniform and the optimizer was AdamW. The CNN architecture was built and the network training was conducted using TensorFlow.[2] As elaborated in the results section of the manuscript, M and N may be varied to suit different applications.

The weights in the connection between the concatenated FCL and FCL32 (enclosed by a grey dashed square) were extracted and analysed to understand the physical significance of structures in the pseudo-PSFs in influencing the learning of the CNN. Further analysis of such weights is provided in Discussion of the main paper and section 5 of this document.

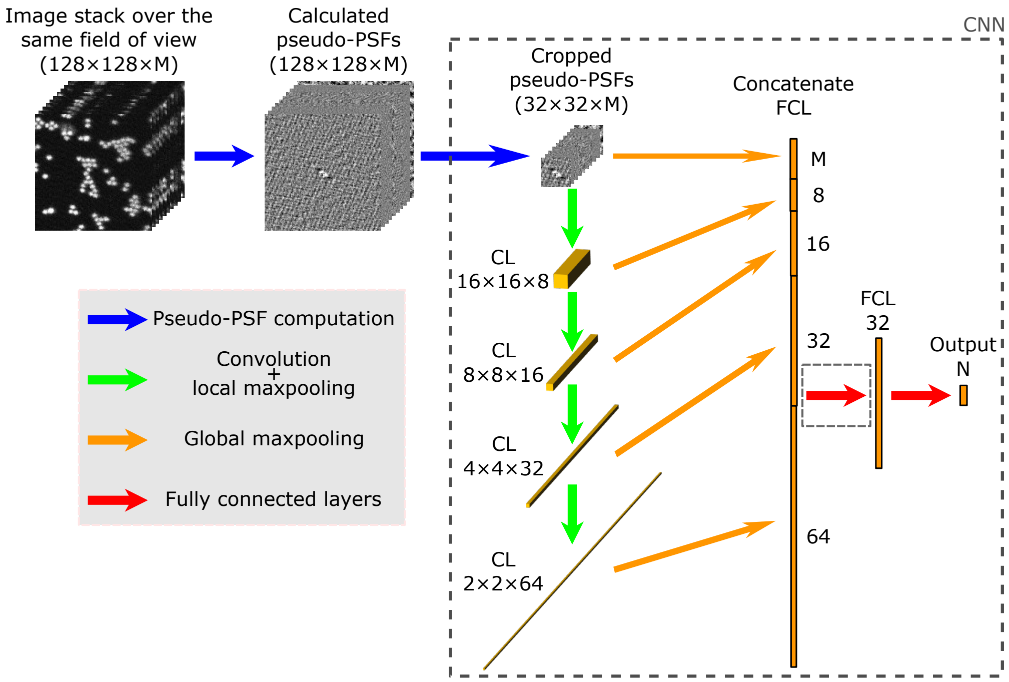

Fig. S1: A schematic illustration of the MLAO process and CNN architecture (enclosed by a black dashed square) designed for phase determination applications. CL: convolutional layer followed by local max-pooling; FCL: fully connected layer; M: number of input images and computed pseudo-PSFs; N: number of estimated output Zernike modes.

2. TRAINING DATA SYNTHESIS

Due to the impracticality of acquiring sufficient high-quality data experimentally, a large dataset of simulated image data was constructed. The simulations were designed to resemble images collected from different microscopes when imaging a range of samples.

We started with a collection of image stacks (containing around a total of 350 images) obtained from high-resolution 3D microscopy of various specimens labelled with nuclear, cytoplasmic membrane and/or single-molecule markers. The images were down-sampled to 8-bit (128×128) and separated into their individual channels. The purpose of down-sampling was to obtain realistic representations of specimen structures containing sharper features that would have been smoothed by the PSF in the original microscope-collected images. This formed a pool of realistic sample structures which were later used to generate synthetic images. To further augment the varieties of sample structures, random rotations were applied and synthetic shapes including dots, rings, circular shapes, curved and straight lines of varying sizes were randomly introduced.

The simulated training dataset was generated by convolving the sample structures with synthetic PSFs, f (see Eq. (1) in the main paper). f was modelled as a pixel array through

$$f = \left| \mathcal{F}\left(P\, e^{j(\Psi+\Phi+\Xi)} \right) \right|^{l} \tag{S1}$$

where $\mathcal{F}$ represented the 2D discrete Fourier transform. P was the circular pupil function, defined such that pixels in the region outside the pupil had value zero. The ratio between the radius of the pupil in pixels and the size in pixels of the overall array was adjusted to match sampling

rates for different microscopes. In practical scanning optical microscopes, the sampling rates can be easily adjusted, although perhaps not arbitrarily. Hence, for experimental flexibility, the ratio for the simulated training dataset was tuned to be within the range of 1.0× to 1.2× the base sampling rate. The base sampling rate was defined as using two pixels to sample the full width half maximum (FWHM) of the PSF of the system when aberration free. For the widefield system, the ratio was tuned to simulate the projection of the camera pixel sampling rate at the specimen. Figure S5 in the supplemental document shows how tolerable a trained network was when tested on data collected at different pixel sampling. P also incorporated the illumination profile for different practical imaging systems, such as when using truncated Gaussian illumination at the pupil in the 3-P microscope. The exponent l varied with imaging modes: when simulating a 3-P, a 2-P and a single photon widefield microscope, l was set to 6, 4 and 2 respectively.

The total aberration was expressed as a sum of chosen Zernike polynomial modes $\Psi + \Phi + \Xi = \sum_i a_i Z_i$. Ψ was the sum of the randomly generated specimen aberrations, which included all modes that the AO system was designed to correct. Φ represented the additional bias aberrations. Ξ included additional non-correctable higher order Zernike modes. The coefficients of the correctable modes were randomly generated for each data set. Representing the set of coefficients $\{a_i\}$ as a vector $\mathbf{a}$, the random coefficients followed a modified uniform n-sphere distribution [3] where both the direction and the two-norm of $\mathbf{a}$ were uniformly distributed. The maximum two-norm (size) of $\mathbf{a}$ were chosen differently for different imaging applications. This distribution allowed a denser population close to zero aberration, which was intuitively beneficial to train a stable NN. We also added random small errors to the correctable coefficients so that the labels were slightly inaccurate. This was to simulate situations when the AO would be incapable of introducing perfect Zernike modes. The spurious high order non-correctable Zernike modes were included to further resemble realistic scenarios in a practical microscope.

Poisson, Gaussian, pink and structured noise of varying noise level were also introduced to the generated images after the convolution to allow the training dataset to simulate more closely real microscope images.

Note that the scalar Fourier approximation of Eq. S1 was chosen for simplicity, although more accurate, vectorial, high numerical aperture (NA) objective lens models could have been applied [4–6]. Although the chosen model would deviate from high NA and vectorial effects, the main phenomena under consideration here – namely the effects of phase aberrations on PSFs and images – are adequately modelled by scalar theory.

3. ZERNIKE POLYNOMIALS AND EXAMPLE PSEUDO-PSFS

A total of ten Zernike polynomials were used for aberration estimation and correction presented in the paper. A list of the polynomials, sequenced using Noll's indices, were included in Figure S2 (a).

Figure S2 (b) included some examples of pseudo-PSFs. It can be observed that when aberration size increases, the maximum pixel value of the Pseudo-PSF decreases; a global max-pooling of the pseudo-PSF extracts information related to the Strehl ratio of the PSFs. Pseudo-PSFs also have shapes that are related to the aberrated PSF shapes.

4. PHYSICAL INFORMATION EMBEDDED IN THE CNN ARCHITECTURE

As mentioned in the main paper, the bespoke CNN architecture embedded information about the physical effects of aberrations on images within the trainable parameters. To illustrate these phenomena, we designed six input patterns and two filters to calculate how values obtained after global max-poolings from different convolutional layers were related to the features of the patterns. Normally, the filters would be learned as part of the training process, but for illustrative purposes, we have defined them manually here.

As shown in Figure S3, patterns 1 to 3 had the same general shape but varying sizes. They were all convolved with the same filter 1. Pattern 1 had the largest feature and the values obtained were almost constant throughout layers 1 to 5 (see Figure S3 (b)). Patterns 2 and 3 had smaller features and the extracted values reduced when moving further down the layers, where the embedded physical scales were more closely related to large scale features. Patterns 4 to 6 had the same general shape with four peaks positioned at the corners of a square. They were all convolved with filter 2, which shared a similar general shape. Pattern 4 had the smallest feature size and resulted a largest value in layer 2. Patterns 5 and 6 had larger feature sizes and resulted in largest values in layers 3 and 4, respectively. This trend confirms the expectation that layers later in the

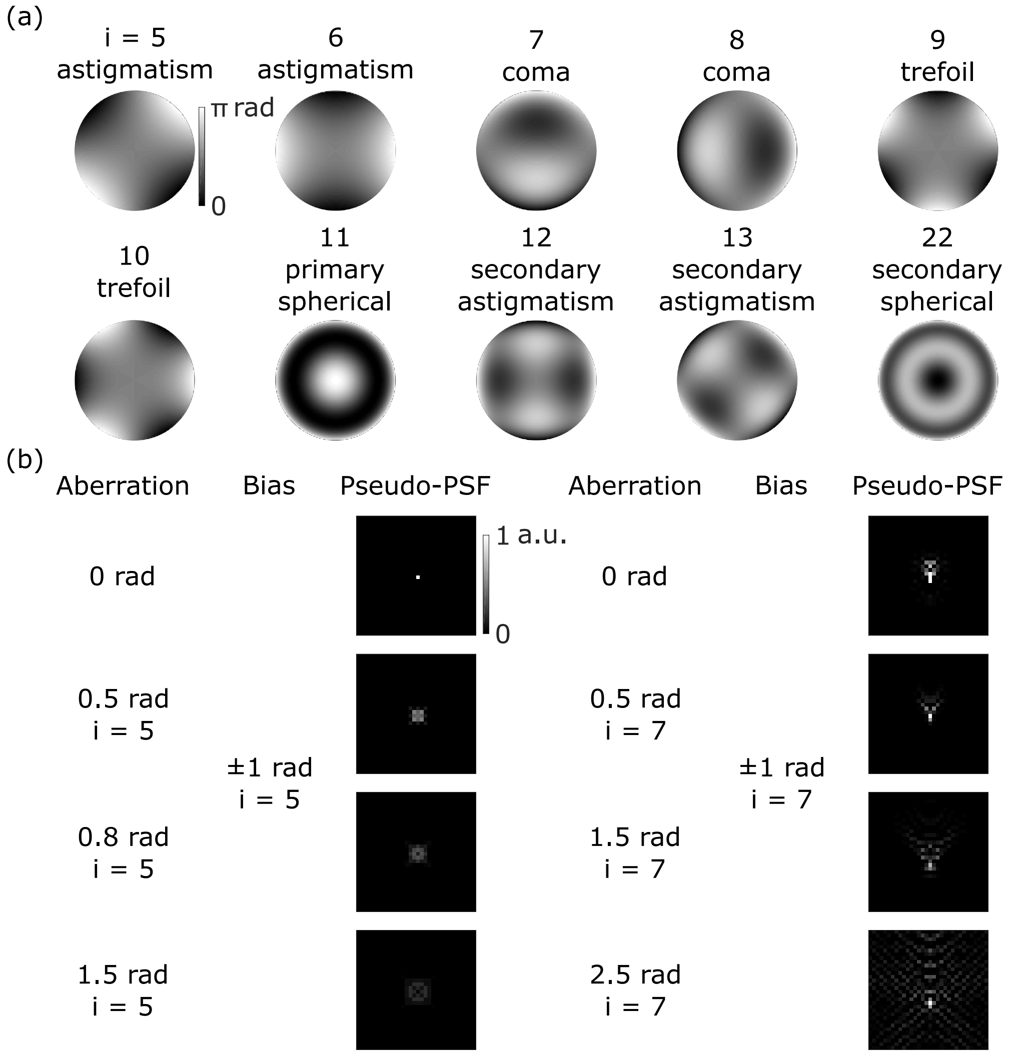

Fig. S2: (a) Zernike polynomials Noll's index 5-13, 22. This is a whole list of the polynomials used for aberration determinations in the paper. (b) Examples of pseudo-PSFs. The first column is the input aberration and the second column is the bias mode used in pseudo-PSFs generation.

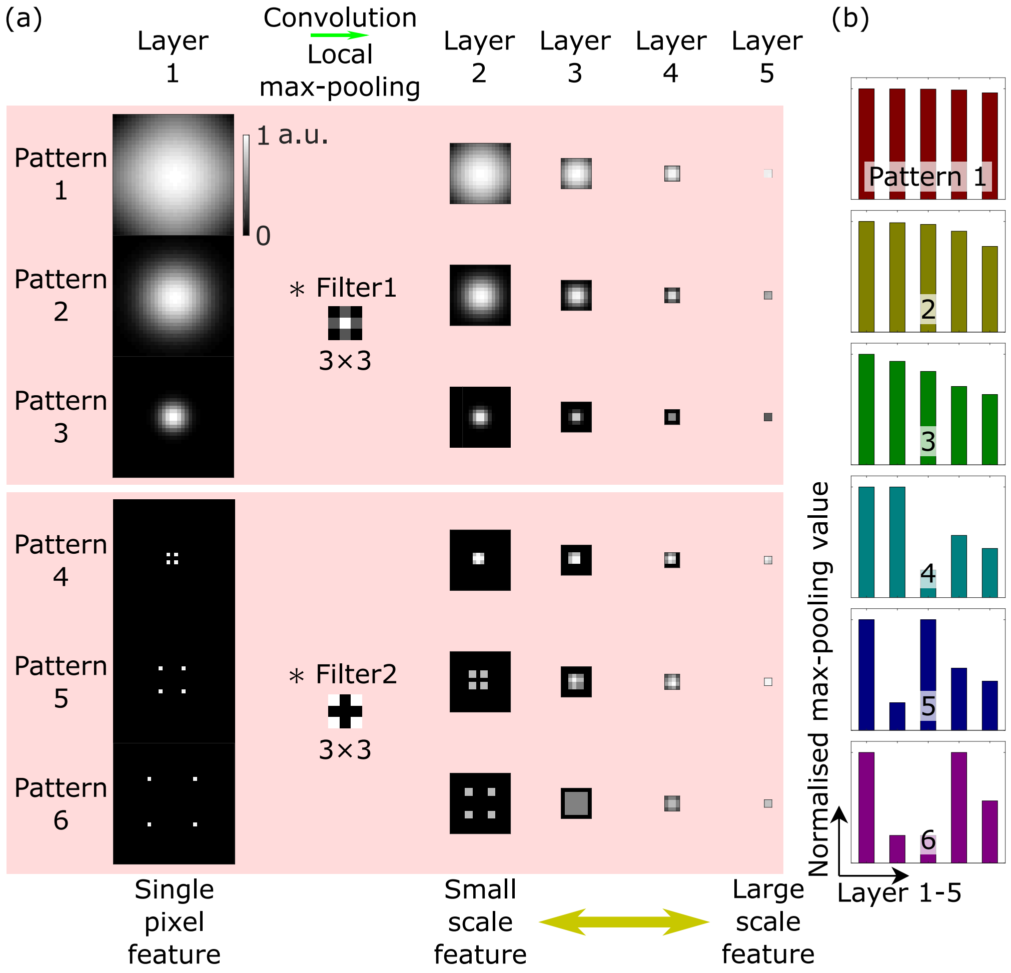

Fig. S3: Demonstrations of the link between feature sizes and convolutional layers. (a) Pattern 1 to 6 each underwent a series of convolutions followed by a 2×2 local max-pooling. Pattern 1 to 3 were convolved with filter 1 and pattern 4 to 6 were convolved with filter 2. For each layer, a global max-pooling were carried out to extract the maximum reading of each layer. The physical interpretations of the extracted values of the different layers were related to Strehl ratio (layer 1) and shapes with features ranging from small scales (layer 2) to large scales (layer 5). The extracted readings was normalised with the readings of their respective previous layer and displayed in (b). The horizontal axis of each plot in (b) indicates from which layer the normalised maximum reading (indicated by the vertical axis) was extracted from.

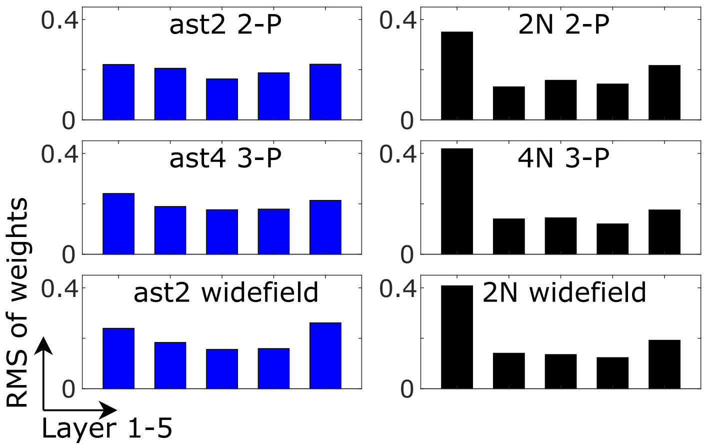

Fig. S4: Analysis of the weight distributions across convolutional layers in the CNNs trained for different biasing schemes and microscopes.

CNN probe larger scales in the input images. Note that all the patterns were designed in such a way that the maximum pixel reading (and thus the value max-pooled from layer 1) equalled to 1.

5. WEIGHT ANALYSIS OF DIFFERENT TRAINED NEURAL NETWORKS

Figure S4 shows the root-mean-square (RMS) values of the weights at the output of each section of the concatenated FCL following the convolutional layers of the CNN. These weights encode information about physical phenomena in the pseudo-PSF that is related to the spatial effects of aberrations on images. Higher numbered layers correspond to larger scale features. Similar distributions are seen for all of the *ast* CNNs class and all of the *2/4N* class. Most notably, it can be seen that the *2/4N* networks all carry heavier weights in layer 1, which is most similar to the Strehl ratio variations of the PSFs.

6. TRAINABLE NEURAL NETWORK PARAMETERS

The bespoke NN and data pre-processing steps were designed with knowledge of the physical basis of image formation. This permitted signficant reduction in NN complexity compared to previous methods for aberration estimation. This architecture not only allowed improved performances, providing insights on internal workings, but also had a structure size orders of magnitude smaller than common NNs used in similar applications (see the comparison in Table S1). This will be beneficial for future applications as NN with fewer trainable parameters would generally require less training data and a shorter training time. Furthermore, the simplified design means that there is greater potential for extending the method to more challenging applications.

Neural network method	Number of trainable parameters
ResNet[7]	>0.27M
Inception V3/ GoogLeNet[8, 9]	23.6M
Xception[10, 11]	22.8M
Deep Image Prior[12]	2M
PHASENET[13, 14]	1M
MLAO in this paper	0.028M to 0.032M

Table S1: A list of NNs used in image processing and phase determination with their number of trainable parameters. Inception V3[8], Xception[10] and PHASENET[13] have been directly demonstrated for phase determination. ResNet is a common basic NN architecture that has been used in many different image processing and phase determination architectures[13]. A 20 layer ResNet is the smallest architecture proposed in the ResNet paper[7] that has ~0.27M trainable parameters. Deep Image Prior employs a U-Net architecture that is a commonly used in many biomedical image processing applications. Deep phase decoder[15], a network designed for wavefront and image reconstruction, was also inspired and adapted from Deep Image Prior.

7. CHOICE OF BIAS MODE

The simplest MLAO implementation uses a pair of biased images as the input. The nature of the bias aberrations is a design choice. In order to investigate this, we tested individual Zernike modes as the bias and trained different MLAO networks with identical architecture to correct the same randomly generated aberrations. The loss function of the different NNs during training was shown in Fig. S5 (a). Results from correcting 20 randomly generated aberrations were shown in Fig. S5 (b).

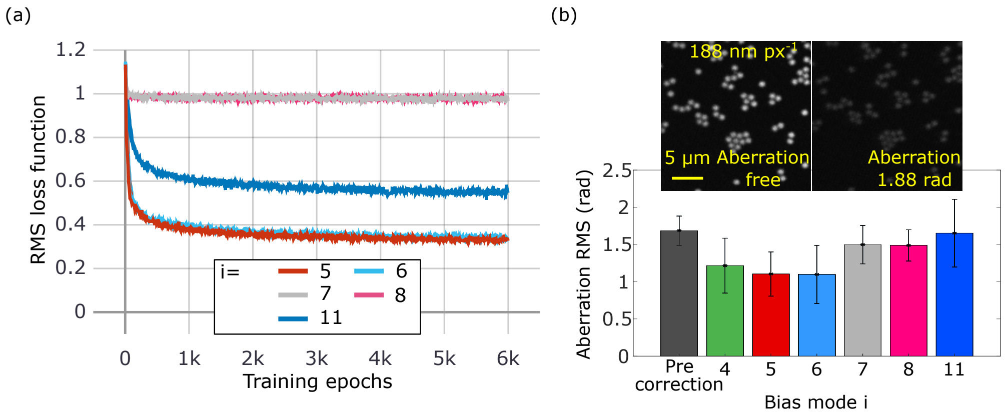

Fig. S5: Testing Zernike modes as choice of bias aberration. (a) A plot of the root mean square (RMS) loss function against the number of epochs when training NNs of the same architecture from the same dataset but using different bias modes. (b) Statistical results of testing the trained NNs to correct the same sets of random aberrations over 2-P microscope images of beads. Twenty randomly generated aberrations consisting five Zernike modes and RMS value smaller than 2.2 radians were introduced for correction (dark gray bar). The remaining aberrations after correction by different networks were averaged and shown in the figure; standard deviations of the remaining aberrations are represented as the error bar. Insets showed an example of the FOV when no aberration was introduced and an example when 1.88 rad of aberration was introduced into the system.

The two networks using oblique and vertical astigmatism (index i =5 and 6) converged to similar loss function during training (Fig. S5 (a)). The same two networks also gave similar averaged remaining aberrations during experimental aberration correction on a bead sample

(Fig. S5 (b)). The two networks using vertical and horizontal coma (index 7 and 8) also showed mutually similar values. This was expected as these pairs of modes (5 and 6; 7 and 8) differ only by rotation, which should not have an effect on how effective the networks determine aberrations.

From these results, the NNs using astigmatism as the bias modes converged to the smallest loss function during training. This possibly suggested that the astigmatism modes, on average, allowed the network to learn more from the training data. It was also observed from the experimental results where, in general, the NN obtained the smallest remaining aberrations. We therefore chose to use astigmatism as the modulation modes for the two-bias NN methods in the experiments conducted in this paper.

8. TOLERANCE TO SAMPLING RATE

As described in the paper, the networks for scanning microscopy were trained on simulated dataset with pixel sampling within the range of 1.0× to 1.2× of the base sampling rate (see the method section in the main paper for more details). However in many practical cases, there can be uncertainty in pixel sampling for a system or constraints on the sampling rates that may be used. We hence tested the tolerance of our networks to pixel sampling rates outside the range of the training dataset (see Fig. S6).

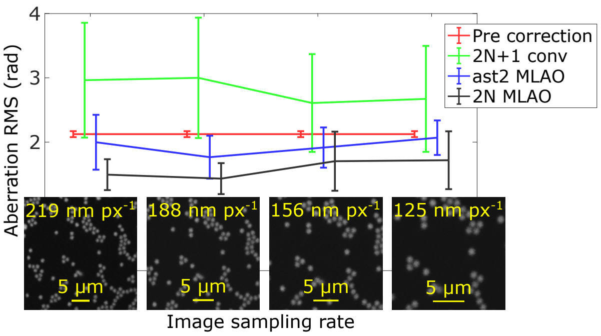

Fig. S6: Testing of robustness to pixel sampling. Statistical results of remaining aberrations before (red plot) and after correction using *2N+1 conv*, *ast2 MLAO* and *2N MLAO* methods. The results were averaged from 20 randomly generated aberrations and the SDs were shown as the error bars. The same algorithms were used to correct the same aberrations over images collected at different pixel sampling as shown by the horizontal axis. Insets show examples of the images collected at different sampling rates.

In this case, 188nm per pixel was close to the sampling of the generated dataset on which the two NNs were trained. When images were sampled at a smaller or larger rate, *ast2 MLAO* and *2N MLAO* were still able to correct aberrations, but were slightly less effective.

9. FURTHER MICROSCOPE DEMONSTRATIONS

Figure S7 showed the performance of the *4N MLAO* and *ast4 MLAO* algorithm, for imaging GFP labelled processes (a) and neuronal activity (b) at a depth of more than 600 μm in a mouse brain when using a 3-P system. Despite the very low SNR of the image data, the image quality and cell activity data were considerably improved.

Figure S8 showed the performance of the *ast2 MLAO* and *2N MLAO* algorithm when imaging neural muscular junctions in a single photon widefield system. The results showed that *ast2 MLAO* corrected much faster than the other two methods.

10. DETAILS OF THE EXPERIMENTAL METHODOLOGY

Three optical systems, a 2-P, 3-P and widefield microscope, were used for demonstrations on different samples. Networks with different parameter settings are also adjusted for different applications.

A. Experimental setups

Figure S9 showed the experimental setups used for the demonstrations in the paper.

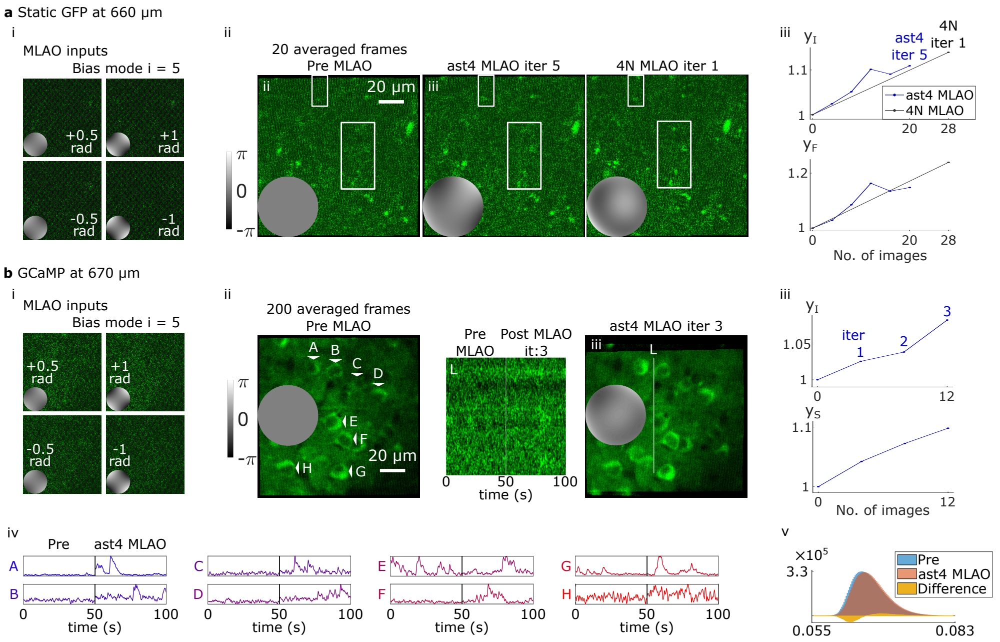

Fig. S7: Three-photon microscopy imaging (a) static GFP at 660μm and (b) GCaMP neuronal activities at depth 670μm. Power at sample was (a) 32.33 mW and (b) 44 mW respectively. Wavefronts inserted to the figures showed the phase modulations applied by the DM at the relevant step; the common scale for each set of results is indicated by the grayscale bars in (a) and (b). (a-i) shows on the left example single-frame images used in correction with the corresponding bias modes as insets; these were the image inputs to *ast4 MLAO*. For *4N MLAO*, six more bias modes and thus 24 more images were also used in each iteration. Three images at the central panel (a-ii) are shown averaged from 20 frames after motion correction. The rectangular boxes highlight regions of interest for comparison. The plots on the right (a-iii) show the intensity metric (y_I) and the Fourier metric (y_F), respectively, calculated from single image frames, against the number of images acquired for five correction iterations of *ast4 MLAO* one correction iteration of *4N MLAO*.

(b-i) shows on the left example single-frame images used as inputs to the *ast4 MLAO* correction with the corresponding bias modes as insets. The central panel (b-ii) shows respectively before and after *ast4 MLAO* correction through three iterations, 200 frame averages after motion correction. The time traces were taken from the marked line L. The plots on the right (b-iii) show the intensity metric (y_I) and the sharpness metric (y_S), respectively, calculated from single image frames, against the number of images acquired for five iterations *ast4 MLAO*. The lower panel (b-iv) shows the calcium activity of 8 cells (A-H marked on the averaged image). The lower right plot (b-v) shows the histograms of the 200 frames collected before (blue) and after (red) *ast4 MLAO* corrections. The difference between the after and before *ast4 MLAO* is marked in yellow. The pixel values are normalised between 0 and 1.

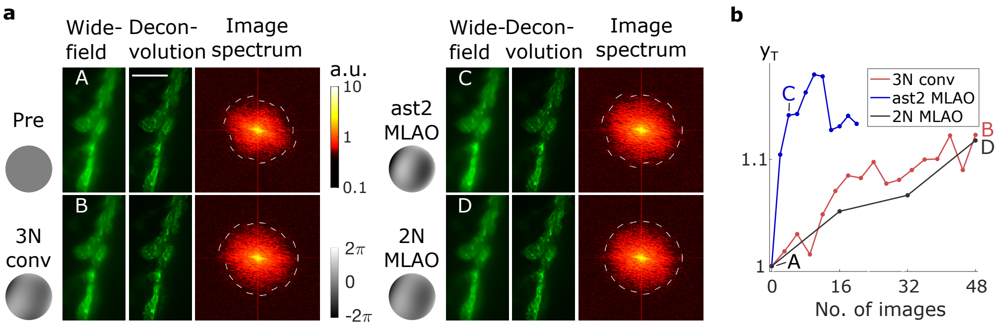

Fig. S8: Aberration correction in a widefield 3-D structured illumination microscope (SIM). (a) Widefield images acquired A before and B-D after correction through different methods (as marked on the metric plot (b)). The first column shows wavefronts corrected by the DM for each image acquisition; phase is shown on the adjacent scale bar. The second column shows the widefield images and the third column shows corresponding deconvolved widefield images. The fourth column shows corresponding image spectra of the second column widefield images displayed in a logarithmic scale (as shown in the colorbar); dashed lines show the threshold where signal falls below the noise level.
(b) The frequency threshold metric y_T against the number of images, for two iterations of *3N conv*, ten iterations of *ast2 MLAO* and three iterations of *2N MLAO*.

B. Sample preparation

The 3-P results were collected from imaging male (Lhx6-eGFP)BP221Gsat; Gt(ROSA)26Sortm32(CAG-COP4*H134R/EYFP)Hze mice (static imaging) and female and male Tg(tetO-GCaMP6s)2Niell mice (calcium imaging). Mice were between 8-12 weeks of age when surgery was performed. The scalp was removed bilaterally from the midline to the temporalis muscles, and a metal headplate with a 5 mm circular imaging well was fixed to the skull with dental cement (Super-Bond C&B, Sun-Medical). A 4–5 mm circular craniotomy was performed during which any bleeding was washed away with sterile external solution or staunched with Sugi-sponges (Sugi, Kettenbach). Cranial windows composed of 4 or 5 mm circular glass coverslips were press-fit into the craniotomy, sealed to the skull by a thin layer of cyanoacrylate (VetBond) and fixed in place by dental cement.

The widefield 3-D SIM results were collected from imaging NMJ of *Drosophila* larvae. For the immunofluorescence sample with one coloured channel, it was prepared as previously [16]. Crawling 3rd instar larvae of wildtype Oregon-R *Drosophila melanogaster* were dissected on a Sylgard-coated Petri Dish in HL3 buffer with 0.3mM Ca2+ to prepare larval fillet [17]. Then, the larval fillet samples were fixed in Paraformaldehyde 4% in PBS containing 0.3% (v/v) Triton X-100 (PBSTX) for 30 minutes. The brains were removed post-fixation, and the fillet samples were transferred to a Microcentrifuge tube containing PBSTX for 45 minutes of permeabilisation. The samples were stained with HRP conjugated to Alexa Fluor 488 and DAPI for 1 hour at room temperature (21C°). After the washes, the samples were mounted in Vectashield.

For the 3-D SIM results collected on the *Drosophila* larvae sample with two coloured channels, it was prepared by following the protocol presented in [16]. 3rd instar *Drosophila* melanogaster larvae (Brp-GFP strain) were dissected in HL3 buffer with 0.3mM Ca2+ to prepare a so-called larval fillet, and the larval brains were removed. After this, larvae were stained for 15 minutes with HRP conjugated to Alexa Fluor 568 to visualise the neurons, washed with HL3 buffer with 0.3mM Ca2+ and imaged in HL3 buffer without Ca2+ to prevent the larvae from moving.

C. Network parameters

Table S2 showed the network settings used in different imaging applications.

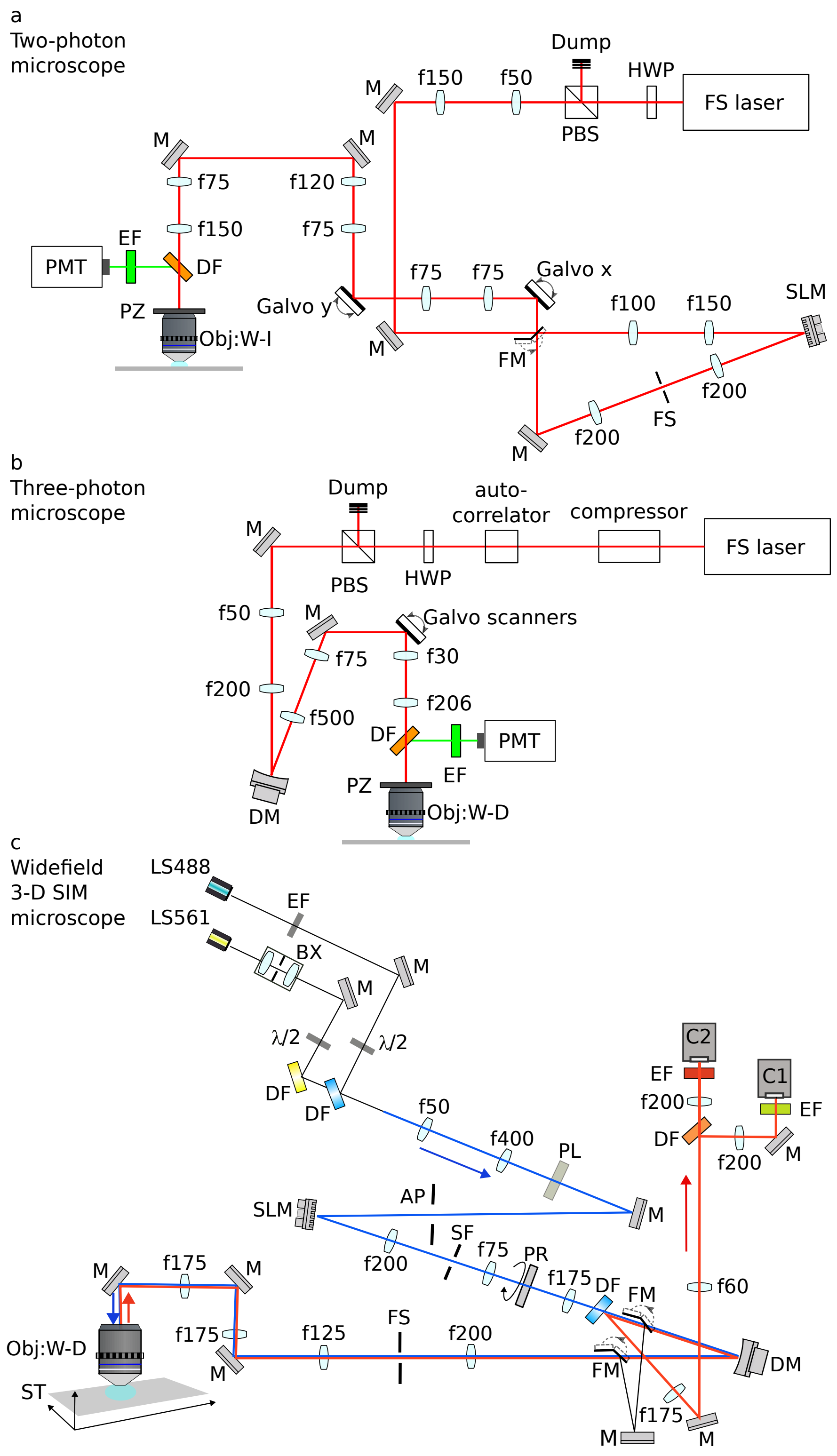

Fig. S9: Configuration of the (a) 2-P (b) 3-P (c) widefield 3-D SIM microscope. (Caption continued on the next page.)

Fig. S9: Femtosecond (FS) Laser; Continuous-wave lasers with wavelenths 488nm and 561nm (LS488 and LS561); half wave plate (HWP); polarisation beam splitter (PBS); laser beam dump (Dump); lens with focal length = x mm (fx); broadband dielectric mirror (M); flip mirror (FM); Hamamatsu spatial light modulator (SLM); Mirao 52E deformable mirror (DM) in the 3-P system; ALPAO 69 deformable mirror (DM) in the widefield 3-D SIM system; aperture (AP); spatial filter (SF); field stopper (FS); X galvanometer (Galvo x); Y galvanometer (Galvo y); beam expansion (BX); half waveplate ($\lambda/2$); linear polariser (PL); polarisation rotator (PR); Olympus 40× numerical aperture (NA) 1.15 water immersion objective lens (Obj:W-I) used in the 2-P system; Nikon 16× NA 0.8 water dipping objective lens (Obj:W-D) used in the 3-P system; Olympus 60× NA 1.1 water dipping objective lens (Obj:W-D) in the widefield 3-D SIM system; Z-piezo translation stage (PZ); X-Y-Z translational sample mounting stage (ST); Dichroic filter (DF) allow emission signal from fluorophores to be reflected through emission filter (EF) into a photo-multiplier tube (PMT) in a multi-photon system; cameras (C1 and C2)

Results in	Method label	M	N	Bias modes, i	Bias depths	Corrected modes, i
Fig. 2 (a, c, f)<br>Fig. S6	*ast2 MLAO*	2	5	5	±1 rad	5–8, 11
Fig. 2 (a, c, f)<br>Fig. S6	*2N MLAO*	10	5	5–8, 11	±1 rad	5–8, 11
Fig. 2 (b, d, e)	*ast2 MLAO*	2	9	5	±1 rad	5–13
Fig. 2 (b, d, e)	*2N MLAO*	18	9	5–13	±1 rad	5–13
Fig. 3 (a, b)<br>Fig. S7	*ast4 MLAO*	4	7	5	±0.5<br>±1 rad	5–11
Fig. 3 (a)<br>Fig. S7	*4N MLAO*	28	7	5–11	±0.5<br>±1 rad	5–11
Fig. 4<br>Fig. S8	*ast2 MLAO*	2	8	5	±1 rad	5–11, 22
Fig. 4<br>Fig. S8	*2N MLAO*	2	8	5–11, 22	±1 rad	5–11, 22

Table S2: A list of MLAO parameters chosen for different imaging applications. The Zernike modes were sequenced using Noll's indices.

REFERENCES

1. Install tensorflow with pip. https://www.tensorflow.org/install/pip
2. Abadi, M. *et al.* TensorFlow: Large-Scale Machine Learning on Heterogeneous Systems. *arXiv preprint*, doi: 10.48550/arXiv:1603.04467 (2016).
3. Marsaglia, G. Choosing a Point from the Surface of a Sphere. *The Annals of Mathematical Statistics* **43**, 645-646, doi: 10.1214/aoms/1177692644 (1972).
4. Ignatowski, V.S. Diffraction by a Lens of Arbitrary Aperture. *Trans. Opt. Inst.* **1**(4), 1-36, doi: 10.1017/9781108552264.019 (1919, Petrograd).
5. Richards, B. & Wolf, E. Electromagnetic diffraction in optical systems, II. Structure of the image field in an aplanatic system. *Proceedings of the Royal Society of London. Series A. Mathematical and Physical Sciences*, doi: 10.1098/rspa.1959.0200 (1959).
6. Boruah, B.R. & Neil, M.A.A. Focal field computation of an arbitrarily polarized beam using fast Fourier transforms. *Optics Communications* **282**, 4660-4667, doi: 10.1016/j.optcom.2009.09.019 (2009).
7. He, K. *et al.* Deep residual learning for image recognition. *Proceedings of the IEEE Conference on Computer Vision and Pattern Recognition*, doi: 10.1109/CVPR.2016.90 (2016).

8. Andersen, T., Owner-Petersen, M. & Enmark A. Neural networks for image-based wavefront sensing for astronomy. *Optics Letters* **44**, 4618–4621, doi: 10.1364/OL.44.004618 (2019).
9. Szegedy, C. *et al.* Going deeper with convolutions. *2015 IEEE Conference on Computer Vision and Pattern Recognition (CVPR)*, doi: 10.1109/CVPR.2015.7298594 (2015).
10. Khorin, P. A. *et al.* Neural networks application to determine the types and magnitude of aberrations from the pattern of the point spread function out of the focal plane. *Journal of Physics: Conference Series* **2086**, 012148, doi: 10.1088/1742-6596/2086/1/012148 (2021).
11. Chollet, F. Xception: Deep Learning with Depthwise Separable Convolutions. *2017 IEEE Conference on Computer Vision and Pattern Recognition (CVPR)*, doi: 10.1109/CVPR.2017.195 (2017).
12. Ulyanov, D., Vedaldi, A. & Lempitsky, V. Deep Image Prior. *2018 IEEE/CVF Conference on Computer Vision and Pattern Recognition*, doi: 10.1109/CVPR.2018.00984 (2018).
13. Saha, D. *et al.* Practical sensorless aberration estimation for 3D microscopy with deep learning. *Optics Express* **28**, 29044-29053 (2020)
14. Saha, D. & Schmidt, U. Phasenet. https://github.com/mpicbg-csbd/phasenet (2020).
15. Bostan E. textitet al. Deep phase decoder: self-calibrating phase microscopy with an untrained deep neural network. *Optica* **7**, 559–562 (2020).
16. Brent, J. R., Werner, K. M. & McCabe, B. D. Drosophila larval NMJ dissection. *Journal of Visualized Experiments* **24**, 1107, doi: 10.3791/1107 (2009).
17. Parton, R. M. *et al.* Drosophila Larval Fillet Preparation and Imaging of Neurons. *Cold Spring Harbor Protocols* **2010**, doi: 10.1101/PDB.PROT5405 (2010).
